# Combination of Clindamycin and Azithromycin as Alternative Treatment for *Toxoplasma gondii* Encephalitis

**DOI:** 10.3201/eid2504.181689

**Published:** 2019-04

**Authors:** Daisuke Shiojiri, Ei Kinai, Katsuji Teruya, Yoshimi Kikuchi, Shinichi Oka

**Affiliations:** National Centre for Global Health and Medicine, Tokyo, Japan

**Keywords:** Toxoplasmic encephalitis, azithromycin, clindamycin, alternative treatment, parasites, HIV, Japan, *Toxoplasma gondii*, meningitis/encephalitis

## Abstract

Current standard therapies for toxoplasmic encephalitis often cause severe adverse events. A 57-year-old HIV-positive man in Japan who had toxoplasmic encephalitis but was intolerant to trimethoprim/sulfamethoxazole, pyrimethamine, sulfadiazine, and atovaquone was successfully treated with the combination of clindamycin and azithromycin. This drug combination can be an alternative treatment for this condition.

Current treatment guidelines for toxoplasmic encephalitis (TE) (*1*) recommend either pyrimethamine plus sulfadiazine or pyrimethamine plus clindamycin; trimethoprim/sulfamethoxazole is also known to have comparable potency (*2*). However, kidney, liver, and hematologic toxicity, as well as hypersensitivities, often have been reported (*2*), and few alternatives are available. We report a TE patient who experienced severe toxicity after all standard regimens but was successfully treated with a combination of clindamycin and azithromycin.

A 57-year-old man sought care at the National Centre for Global Health and Medicine (Tokyo, Japan) with fever and mild disorientation. On examination, he was febrile and drowsy with slurred speech. He did not exhibit any apparent focal neurologic deficits. 

Blood laboratory test results (reference ranges) were as follows: hemoglobin, 10.4 g/dL (13.7–16.8 g/dL); total leukocyte count, 6.81 × 10^3^ cells/L (3.3–8.6 × 10^3^ cells/L); platelet count, 2.43 × 10^5^/L (1.58–3.48 × 10^5^/L); β-d-glucan, 118.4 pg/mL (<20.0 pg/mL); CD4^+^ count, 58 cells/μL (500–1,500 cells/μL); and HIV RNA, 5.1 × 10^5^ copies/mL (undetected). Brain magnetic resonance imaging results indicated 3 lesions with high intensity on T2-weighted images and irregular ring enhancement. The lesions were in the right frontal lobe (diameter 15.6 mm), left globus pallidus (diameter 17.5 mm), and body of the caudate nucleus extending to the left thalamus (diameter 16.2 mm; Figure, panel A). For cerebrospinal fluid (CSF), laboratory test results (reference ranges) were as follows: leukocyte count 0.3 cells/mm^3^ (0–5 cells/mm^3^); protein, 39 mg/dL (15.8–52.6 mg/dL); glucose, 54 mg/dL (47–69 mg/dL); and adenosine deaminase, 2.2 U/L (0–20 U/L). Results of real-time PCR were negative for *Mycobacterium*, Epstein–Barr virus, cytomegalovirus, herpes simplex virus, and JC virus in CSF. Despite mild elevation of β-d-glucan, results of cryptococcal antigen testing were negative. No brain biopsy was performed. Although serum toxoplasma IgM and IgG titers were initially negative, IgG seroconversion was observed in serial tests 6 months (9.2 IU/mL) and 12 months (39.7 IU/mL) later. Toxoplasma nucleic acid in CSF was detected using a loop-mediated isothermal amplification technique (LAMP) with previously validated methods (*3*).

**Figure:**
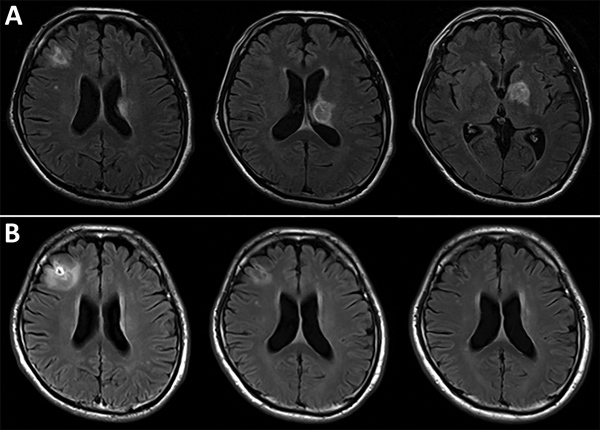
Serial brain magnetic resonance imaging results for a 57-year-old man with *Toxoplasma gondii* encephalitis, Tokyo, Japan. A) All 3 lesions were evident when the patient first sought care. B) Chronologic changes are shown of the lesion in the right frontal lobe in response to antitoxoplasmic therapy after 1 (left), 3 (center), and 12 (right) months.

Trimethoprim/sulfamethoxazole (10 mg/kg/d trimethoprim) was first initiated because of limited availability of pyrimethamine plus sulfadiazine at the time, but acute liver toxicity developed on treatment day 6 (increases in levels of aminotransferase/alanine aminotransferase from 24/20 IU/L to 127/145 IU/L[(reference 10–42 IU/L]). Pyrimethamine plus sulfadiazine was started on treatment day 7. However, on day 9, acute renal failure developed because of obstructive urolithiasis caused by sulfadiazine crystals. After the patient was switched to pyrimethamine plus clindamycin, his renal function persistently worsened, and a drug-induced lymphocyte stimulation test suggested that pyrimethamine was responsible for the acute renal failure. A switch to atovaquone on treatment day 12 resulted in thrombocytopenia by day 17.

After switching to the combination of clindamycin (2,400 mg/d) and azithromycin (1,200 mg/d), the patient did not experience any apparent adverse effects or show abnormal laboratory values. He successfully started antiretroviral therapy for his HIV infection during week 6. Repeated brain magnetic resonance imaging performed 1, 3, and 8 months after presentation showed all lesions had substantially decreased, and only a residual nonenhancing lesion was observed 12 months after presentation (Figure, panels B–D). The patient has continued maintenance therapy with the same dose of clindamycin and azithromycin without any symptoms of relapse or immune reconstitution inflammatory syndrome.

Several reports have documented the high prevalence of various adverse events associated with the aforementioned standard therapies for TE (*2**,**4*), but little information is available regarding the efficacy of alternative drugs. Although atovaquone is the only safe alternative agent for which efficacy and safety have been relatively well established, it has been proven to be less potent than standard therapies (*5*).

Clindamycin has in vitro activity against *Toxoplasma gondii*, inhibitory effects in vivo at lower concentrations (*6*), and the ability to penetrate into CSF (*7*), suggesting that it is a promising alternative agent for TE treatment. More important, clindamycin causes far fewer adverse events than sulfadiazine (*2*,*4*). Although the potency of clindamycin monotherapy has not been assessed, some case reports suggested its effectiveness (*8*). Therefore, a clindamycin-containing combination with potent agents other than pyrimethamine may be a reasonable treatment option for TE treatment.

Macrolides also have displayed substantial in vitro efficacy against *T. gondii* (*9*). A phase I/II dose-escalation study of oral azithromycin in combination with pyrimethamine revealed the relative efficacy and safety of the regimen (*10*). Azithromycin combined with a potent agent other than pyrimethamine, such as clindamycin, can be theoretically expected to be a safe and effective option. Further study should be conducted to establish safer treatment options for TE. 

The continuous reduction in the size of the lesion in the patient we report was partly explained by his good immune recovery. Our results suggest that the combination of clindamycin and macrolides could be a safer and potent alternative therapy for patients who are intolerant of current standard regimens.
